# Tumor response to radiotherapy is dependent on genotype-associated mechanisms in vitro and in vivo

**DOI:** 10.1186/1748-717X-5-71

**Published:** 2010-08-12

**Authors:** Jerry R Williams, Yonggang Zhang, Haoming Zhou, Daila S Gridley, Cameron J Koch, John F Dicello, James M Slater, John B Little

**Affiliations:** 1 Radiation Research Laboratories, Department of Radiation Medicine, Loma Linda University Medical Center, Loma Linda CA, USA; 2 Laboratory of Radiobiology, Johns Hopkins School of Medicine, Baltimore, MD, USA; 3 Department of Radiation Oncology, University of Pennsylvania, Philadelphia, PA, USA; 4 Center for Radiation Sciences and Environmental Health, Harvard School of Public Health, Boston, MA, USA

## Abstract

**Background:**

We have previously shown that in vitro radiosensitivity of human tumor cells segregate non-randomly into a limited number of groups. Each group associates with a specific genotype. However we have also shown that abrogation of a single gene (p21) in a human tumor cell unexpectedly sensitized xenograft tumors comprised of these cells to radiotherapy while not affecting in vitro cellular radiosensitivity. Therefore in vitro assays alone cannot predict tumor response to radiotherapy.

In the current work, we measure in vitro radiosensitivity and in vivo response of their xenograft tumors in a series of human tumor lines that represent the range of radiosensitivity observed in human tumor cells. We also measure response of their xenograft tumors to different radiotherapy protocols. We reduce these data into a simple analytical structure that defines the relationship between tumor response and total dose based on two coefficients that are specific to tumor cell genotype, fraction size and total dose.

**Methods:**

We assayed in vitro survival patterns in eight tumor cell lines that vary in cellular radiosensitivity and genotype. We also measured response of their xenograft tumors to four radiotherapy protocols: 8 × 2 Gy; 2 × 5Gy, 1 × 7.5 Gy and 1 × 15 Gy. We analyze these data to derive coefficients that describe both in vitro and in vivo responses.

**Results:**

Response of xenografts comprised of human tumor cells to different radiotherapy protocols can be reduced to only two coefficients that represent 1) total cells killed as measured in vitro 2) additional response in vivo not predicted by cell killing. These coefficients segregate with specific genotypes including those most frequently observed in human tumors in the clinic. Coefficients that describe in vitro and in vivo mechanisms can predict tumor response to any radiation protocol based on tumor cell genotype, fraction-size and total dose.

**Conclusions:**

We establish an analytical structure that predicts tumor response to radiotherapy based on coefficients that represent in vitro and in vivo responses. Both coefficients are dependent on tumor cell genotype and fraction-size. We identify a novel previously unreported mechanism that sensitizes tumors in vivo; this sensitization varies with tumor cell genotype and fraction size.

## Introduction

Much research in clinically-relevant radiobiology is based on the premise that there is a triangular relationship between radiocurability of tumors in the clinic, radiosensitivity of xenograft tumors in vivo and radiosensitivity of human tumor cells in vitro. We have previously reported, in collaboration with Vogelstein's laboratory, that abrogation of a single gene (p21) increases susceptibility of xenograft tumors to radiotherapy but compared to its parent line, does not effect in vitro radiosensitivity [1]. This was the first report showing modulation of a single gene could uncouple in vitro versus in vivo radiosensitivity. It also implies that in vitro radiosensitivity alone cannot predict tumor response.

We now compare in vitro and in vivo responses of multiple human tumor cells that vary in radiosensitivity and genotype. We selected a set of human tumor cells from a large study that defined radiosensitivity as measured in vitro. These cell lines segregated into radiosensitivity groups and each group associated with genotype, not histological type [2,3]. When these data are placed in an appropriate structure, tumor cell radiosensitivity segregates into distinct groups that each associate with a specific genotype. Four genotypes were identified that were markers for these radiosensitivity groups: mutant ATM, wildtype TP53, mutant TP53 and an unidentified gene or factor (glio) that renders a subset of glioblastoma cells very radioresistant [2,3]. These cell lines represent the most sensitive cell line we have examined (SW1222), the most resistant cell lines we have examined (U251) and six cell lines that represent the most common genotypes expressed in human tumor cells, wtTP53 and mutTP53. We now define in vivo radiosensitivity of xenograft tumors comprised of these cell lines that represent these four cellular radiosensitivity groups. We stress that while we selected cell lines from each radiosensitivity group, we did not select specific genotypes. Oncogenesis selected the four genotypes that segregate with tumor radiosensitivity.

Critical to interpreting our data is confidence that xenograft tumors reflect relevant properties of cellular radiosensitivity. Xenograft tumors have been demonstrated to be a useful general tool for studying in vivo radiosensitivity compared to in vitro characteristics of their constituent cells [4-6]. Xenograft studies have been particularly useful in studying the dose-rate effect [7], the effect of dose-fractionation [8,9] identification of the α/β ratio [10] and the role of TP53 in tumor response [11]. Xenograft studies have been used to seek correlations between in vitro and in vivo response for tumors of different histological types, including melanoma [12], breast [13], lung [14], colon [15], glioblastoma [16] and squamous cell carcinoma [17]. We have previously used xenograft studies to show abrogation of a single gene, CDKN1A (p21), increases xenograft tumor radiosensitivity to large fractions (15 Gy) in vivo but does not alter cellular radiosensitivity in vitro [1]. Similarly some genomic manipulations increase sensitivity to other anti-cancer agents but not ionizing radiation [18].

Multiple methods have been used to describe quantitative response of xenograft tumors to radiotherapy. For instance the use of TCD_50 _(mean dose required to inhibit regrowth in 50% of tumors) is a powerful yet resource-intensive method [19]. We and others have used direct comparison of kinetics of regrowth delay between pairs of tumor types or between pairs of radiotherapy protocols [1,18] and while this method has significant statistical power in such a pair-wise comparison, it is limited in comparing response of multiple tumors that vary widely when irradiated with different radiotherapy protocols. We now study the response of multiple cell lines that vary extensively in genotype and susceptibility to cell killing in vitro, for the relative sensitivity of their xenograft tumors in vivo. It was important to measure tumor response over a wide range of cell and tumor sensitivities so we selected a modification of the method of Schwachofer et al [20] to describe tumor response to radiotherapy based on modal volume of regrowing tumors even when some tumors do not regrow. These methods are described below.

## Materials and methods

### Cell and culture techniques

Human colorectal tumor cell lines (HCT116, 80S4, 14-3-3σ-/-, 379.2, DLD1 and 19S186) were obtained from Dr. B. Vogelstein of the Oncology Center of Johns Hopkins, School of Medicine), SW1222 was from Dr. James Russell (Memorial Sloan-Kettering Cancer Center, NY), and U251 was purchased from ATCC. The basic media for all colon tumor cell lines was McCoy 5A, supplemented with 10% FBS, 1% penicillin and streptomycin, 1% L-glutamine; 14-3-3σ-/- required addition of G418 (0.5 mg/ml); SW1222 was grown in RPMI 1640. Human glioma cell line U251 was cultured in DMEM/F12 with 10% FBS, 1% L-glutamine and 1% Penicillin and streptomycin. All cells were sub-cultured twice a week to maintain exponential growth.

### Cell survival assay

Cells were plated ~18 hours before irradiation. Surviving colonies were determined 10-14 days after irradiation depending on the cell line. Cells were stained with crystal violet and colonies counted (>50 cells/colony). Additional plates for each experiment were used as microcolony controls.

### Radiation treatment

Cells were irradiated using a ^137^Cs AECL Gammacell40 gamma irradiator at 0.7 Gy/min. For irradiation of xenograft tumors, mice were confined in 50 ml plastic centrifuge tube with holes through which the tail and the tumor-bearing leg could be extended. Tumors were irradiated at dose rate of 7.5 Gy/min with a collimated beam in a J.L. Shepard Mark I ^137^Cs irradiator (Pasadena CA USA).

### Tumor growth delay assay

Tumors were established by subcutaneous injection of 5 million cells suspended in PBS into the upper thigh of nude mice. Each cohort included 6 to 13 tumors. Tumor growth rate was determined by measuring three orthogonal diameters of each tumor twice a week and the tumor volume estimated as π/6[D1 × D2 × D3], when individual tumor volumes reached ~0.1-0.3 cm^3^, radiation treatment was initiated. Modal specific growth delay (mSGD) was measured for all cohorts in which a majority of tumors reached a volume four times the initial volume. Response was normalized to growth of unirradiated cells. We chose not to use the mean of specific regrowth delay patterns since a significant proportion of our cohorts included one or more tumors that did not regrow. Thus the mean became limited as a regrowth parameter. In our forty xenograft experiments, only cohorts of the very sensitive (VS) cells, SW1222, less than half the tumors regrew when treated with 7.5 and 15.0 Gy and thus the modal values for SGD are no longer meaningful. For these two cohorts we estimated mSGD based on the regrowth pattern for the minority of tumors that did regrow. When we tested the sensitivity of modal to mean growth delay in selected cohorts in which all tumors regrew, the modal value always fell within one standard deviation of the mean. These methods share some characteristics of the methods described by Schwatchofer [20]. To provide an overview of the dichotomous response when some tumors regrow but some do not, we indicated such cohorts with an arrow showing this value, in terms of overall tumor response, was the common minimum response.

### Statistical analysis

Comparison of data clusters were evaluated using Student's t test with p < 0.05 as the level for significance.

## Results

Our data are presented as three major observations: 1) In vitro radiosensitivity of tumor cells and in vivo radiosensitivity of their xenograft tumors show specific relationships that vary with genotype; 2) this large data matrix can be structured into an analytical system based on two coefficients that describe in vitro and in vivo radiosensitivity in parametric terms; and 3) these comparisons demonstrate a new heretofore unrecognized mechanism that influences in vivo radiosensitivity.

We selected eight cells from the four in vitro radiosensitivity groups and these cell lines are shown in table 1. In this table we list these lines by radiosensitivity groups, by histological type, comments on their molecular characteristics, and comments on their radiosensitivity. This table also shows their expression of DNA mismatch repair enzymes, homozygous deficiency in such genes suggest the tumor developed in individuals that express the genetic syndrome HNPCC (Human Non-Polyposis Colorectal Cancer).

**Table 1:** Genetic variation and in vitro radiosensitivity of eight human tumor cell lines.

Radio-Sensitivity Group*	Cell Line	Genetic Characteristics			In Vitro Radiosensitivity
		TP53	p21 induced	MMR	
VR	U251	mt (273arg-his)	-	+	Most resistant cell line, other radioresistant glioblastomas segregate into this group.
R	DLD1	mt (241ser-phe)	-	hMSH6-	Other epithelial tumors that express mutTP53 segregate into this group.
	19S186	p21 double knockout from DLD1			
S	HCT116	wt	+	hMLH1-	Other epithelial tumors that express wtTP53 segregate into this group.
	379.2	p53 double knockout from HCT116.			
	80S4	p21 double knockout from HCT116			
	14-3-3σ-/-	14-3-3σ double knockout from HCT116			
VS	SW1222	null	-	+	Most sensitive cell line, mutant in the ATM gene with an A moiety inserted in codon 6997 of exon 50.

As defined in Williams et al. [2].

Cell lines fall into four radiosensitivity groups as defined by Williams et al. 2007, 2008a. All cell lines were derived from human colorectal tumors except U251 that is derived from a human glioblastoma. Expression of TP53 and radiation induced p21 were assayed by Western blot analysis. Deficiency in MMR (DNA mismatch repair) is a marker that these tumor developed in individuals expressing HNPCC (human non-polyposis colorectal cancer).

### In vitro radiosensitivity

**Figure 1:**
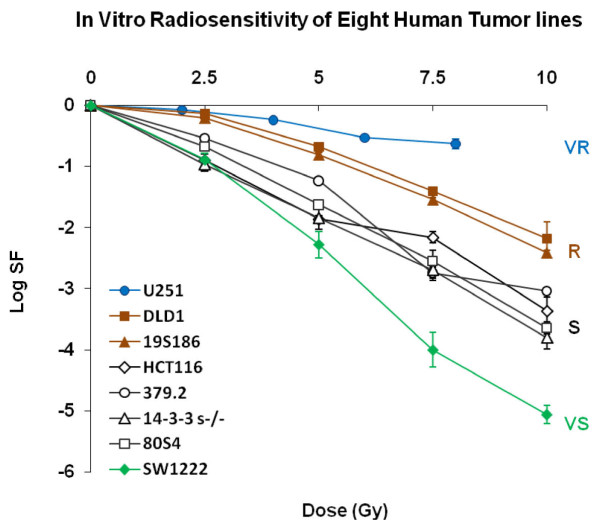
**Clonogenic survival for eight human tumor cells lines described in table 1**. Data points are the mean and standard deviation for 3 to 5 replicates. Four radiosensitivity groups are designated as VR, R, S and VS as defined in reference 5.

We irradiated each of the eight cell lines in table 1 with graded doses of ionizing radiation and measured colony formation. These data are shown in figure 1.

These data represent the range of human tumor cell radiosensitivity as observed across a large cohort of human tumor cells. Each radiosensitivity group expresses a common genotype and each clonogenic inactivation in each group is statistically distinct at circa 2 Gy. However the distribution of tumor cell radiosensitivity with genotype is better seen when radiosensitivity of tumor cells is expressed as the ratio of radiosensitivity at circa 2 Gy and radiosensitivity at higher doses. In references [2,3] we have designated the four cellular radiosensitivity groups as VS (very sensitive), S (sensitive), R (resistant) and VR (very resistant) based on statistical differences in survival at 2 Gy. The four groups of tumor cells are statistically different in survival levels at circa 2 Gy. However the overall relationship between genotype and in vitro radiosensitivity is better illustrated when shown as correlation between two slopes that represent clonal inactivation over two dose ranges.

**Figure 2:**
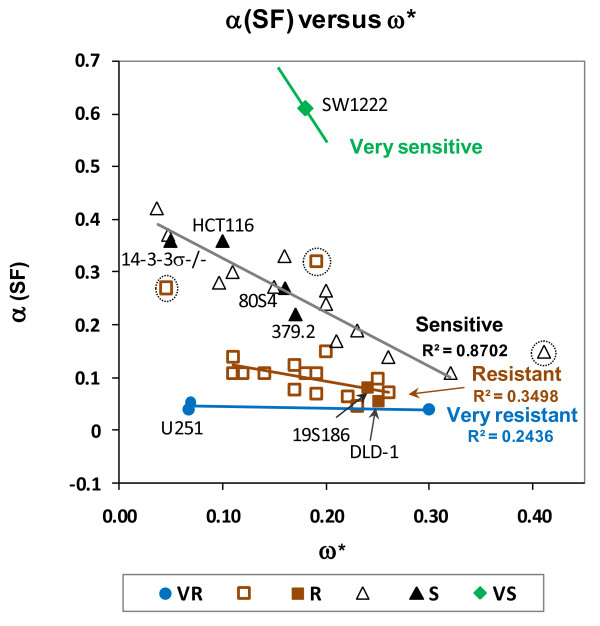
**In vitro cellular radiosensitivity of eight cell lines used in figure 1 presented within a data matrix representing the spectrum of tumor cell radiosensitivity**. Data are expressed as the ratio of coefficients that describe the slope of clonogenic inactivation at lower doses α(SF2) and ω*, the rate of additional clonogenic inactivation at higher doses.

We show these data in figure 2 for survival data in figure 1 placing radiosensitivity of these ten cell lines in a structure of coefficients that describe their radiosensitivity within a framework of radiosensitivity for 39 cell lines. Radiosensitivity of each cell line is expressed as defined by the ratio of cell killing at circa 2 Gy, α (SF2) to additional cell killing at doses higher than 4.0 Gy, ω*. This figure shows the relative cellular radiosensitivity of the eight cells used in the experiments present as four diagonal lines, each line associated with a specific genotype.

These data are shown in figure 2 as four linear arrays, each array comprised of a radiosensitivity group that share genotype. Most human tumor cell lines established from clinical specimens fall into two radiosensitivity groups, S and R. Tumor cells that fall into the S radiosensitivity group express predominantly, but not exclusively, wtTP53. Indeed a cell line (379.2) that has been abrogated in TP53 as a mature cancer cell, shares the S response even though null for TP53 expression. The S cell group also includes sublines of the colorectal tumor line HCT116 that have been abrogated in CDKN1A, p21 (80S4 cells) or abrogated in 14-3-3 σ (14-3-3σ-/-). 80S4 cells (p21-) are from the cell line that we showed have increased radiosensitivity as xenograft tumors [1]. The R radiosensitivity group is comprised predominantly, but not exclusively, of cells that express mutTP53. In our studies the R radiosensitivity group is represented by DLD-1 that expresses mutTP53 and one subline that has been abrogated in CDKN1A, p21 (19S186). VS cells (SW1222 cells) are mutant in ATM (an A moiety inserted in codon 6997, codon 50) and this is the most sensitive cell line we have identified. A VR cell line (U251 cells) is representative of the most radioresistant group of human tumor cells. Importantly, four cell lines in figures 1 and 2 show diminished levels of p21 expression: 80S4 cells, that represents abrogation of p21 in a wildtype TP53 background; 19S186 cells represent abrogation of p21 in a mutant TP53 background; the cell line mutant in ATM and the radioresistant glioblastoma line. The data in figures 1 and 2 show that abrogation of p21, 14-3-3σ and surprisingly TP53 does not modulate in vitro radiosensitivity. The fact that abrogation of TP53 does not shift radiosensitivity from the S group demonstrates that the presence of wtp53 protein is not involved in the expression of S radiosensitivity observed in all cells that express wildtype TP53.

### In vivo radiosensitivity of xenograft tumors comprised of cells that vary in their in vitro radiosensitivity and genotype

**Figure 3:**
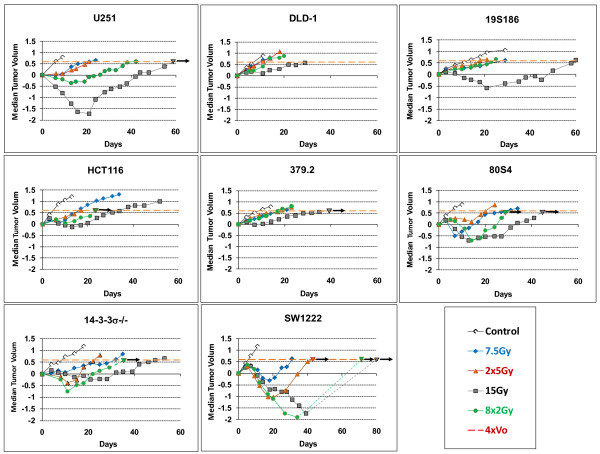
**Relative tumor volume as a function of time after irradiation for eight tumor cell lines responding to five protocols**. Tumor volume is expressed as the log of the ratio of the volume of irradiated cells compare to unirradiated cells at specific post-irradiation times. Each panel represents response of one of eight cell lines to five different treatment protocols as shown in the legend. Data points are the modal values of 6 to 13 tumors. Where all tumors did not regrow there is an arrow above the final point that indicates modal value was measured using only the tumors that regrew. The two responses for SW1222 cells at 8 × 2 and 1 × 15 show a dotted line where the value for modal Specific Growth Delay are portrayed using less than a majority of tumor the did regrow.

For each of the eight cell lines for which we determined in vitro radiosensitivity in figures 1 and 2, we measured in vivo radiosensitivity of their xenograft tumors. Five cohorts of xenograft tumors comprised of 6 to 13 tumors from each cell line were exposed to five different protocols. These protocols are: control; two single dose protocols: 1 × 7.5 Gy and 1 × 15.0 Gy; and two fractionated protocols: 8 × 2 Gy, with fractions of 2.0 Gy each delivered over three days with at least 6 hours between fractions and 2 × 5 Gy, delivered with 24 hours between fractions. Radiation-induced changes regrowth of human tumors for these 40 cohorts of tumors are shown in figure 3.

These data, representing over 3000 individual data, show an extremely wide range of in vivo radiosensitivity for different genotypes on the basis of protocols. Certain general observations can be made before detailed analysis. First, response of tumors comprised of SW1222 (mutATM) cells are hypersensitive to all protocols, both fractionated and acute. Total dose dominates responses of this cell lines and sparing by fractionation is not as effective as other cell lines. Surprisingly the most resistant cell line U251 is unexpectedly sensitive to larger fractions. In general cells from the R group are more resistant over most protocols compared to the S group.

The wide range of data in this figure demonstrates how the use of modal SGD allows estimation of a single parameter over all cell types and protocols. Only for two cohorts, VS cells treated with 15 Gy acute or 16 Gy delivered as 8 fractions, did fewer than half the tumors failed to regrow shown as terminal values observed at day 34 for the 8 × 2 Gy cohort and at 40 days for the 1 × 15 Gy treatments. In figure 3, these cohorts we draw a dotted line representing the response of the tumors that did regrow but constituted less than half the total tumors in the cohort.

To indicate the effect of dichotomous response, wherein all tumors in a cohort did not regrow, we indicate these with a short arrow at the value of mSGD where measurements are made.

### Tumor regrowth delay varies extensively with irradiation protocols and tumor genotype

Four cell lines in figure 3 show exceptional levels of regrowth delay after irradiation with single fractions of 15 Gy and these are: SW1222 (mutATM), 80S4 (wtp53, p21-), 19S186 (mutTP53, p21-) and U251 (radioresistant glioma "glio"). Based on our previous work [1] we expected this elevated response for tumors comprised of cells abrogated in p21(80S4 cells, p53+, p21-) and perhaps for SW1222 (mutATM) cells that have exceptional radiosensitivity in vitro, but the response of 19S186 cells (mutp53, p21-) and especially the response of U251 cells (glio) were not expected. On the basis of this clear dichotomy in response to 15Gy expressed by tumors comprised of four cell lines compared to the other four lines we will present and analyze our data on the basis of two response groups, one designated the "S-R response group" and postulate it represents most cell lines that fall into the S and R radiosensitivity groups. The other group will be identified at this point as "p21^- ^response group" and includes two cell lines abrogated in p21 (80S4 and 19S186) and two cell lines shown in table 1 to express diminished p21 (SW1222 cells and U251 cells).

### Development of an analytical structure to compare in vitro and in vivo radiosensitivity

In the next several figures we propose a simple analytical structure that can be used to compare in vitro and in vivo radiosensitivity.

### Expressing the overall relationship between total dose and tumor response

**Figure 4:**
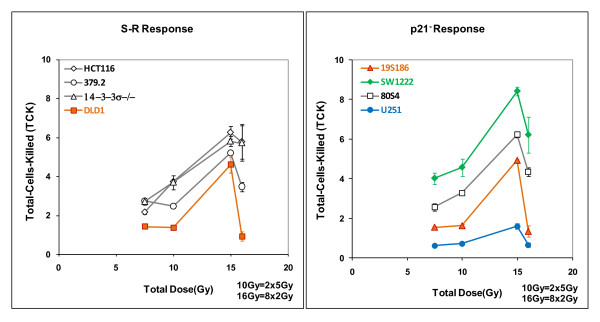
**Overall tumor response, expressed as modal specific growth delay in days, derived from figure 3 plotted against total-cell-killing derived from figure 1**. Each cell line is represented by four responses shown as two responses to fractionated doses (8 × 2 Gy and 2 × 5Gy) connected by dotted lines and two responses to single acute fractions (1 × 7.5 Gy and 1 × 15.0 Gy) connected by solid lines. Data in the left hand panel shows responses of the S-R cells fall into a common linear pattern with a correlation coefficient of R^2 ^= 0.7271. The data in the right hand show four lines response in a relatively linear pattern (R^2 ^= 0.7271) but the cell lines in the right hand panel do not The best fit correlation line for the data in the left hand panel is shown as a solid line on that panel and also redrawn on the right hand panel for comparison. The trapezoid in the right hand panel includes all data from the left hand panel, emphasizing the differences in scale between the two panels. Data points are individual measurements.

The data in figures 1, 2 and 3 can be used to determine the relationship between tumor response expressed as mSGD and total-cells-killed (TCK) expressed as logs of tumor cells inactivated. When we performed this analysis we observed two distinct patterns each observed in two groups of cell lines. In figure 4 and subsequent figures we will present a parallel analysis of these two groups. This dichotomy is based on distinct differences in tumor response as a function of total dose. These data are shown in figure 4.

These data show that tumor genotype influences response of xenograft tumors to radiotherapy. These data segregate data into two different response patterns. The correlation between xenograft responses for four genotypes shown in the left hand panel is a relatively linear relationship between tumor response and log of total-cells-killed but the xenografts responses for four other genotypes as shown in the right hand panel, are distinctly elevated. For both panels, the arrows pointing to the right indicate that modal Specific Growth Delay was determined by the majority of tumors in the cohort but that one or more tumors did not regrow. Thus the data points with arrows are an estimate of minimum regrowth delay.

The data in the left hand panel show relatively strong correlation between tumor response and logs of total-cells-killed with a relatively high correlation coefficient of 0.7271, a surprisingly strong correlation for data derived from multi-factor biological experiments. We will refer to this group for the benefit of discussion as the S-R tumor radiosensitivity group as the tumors in this panel are comprised of four cell lines from the S and R cellular radiosensitivity groups. The four genotypes that fall into the more linearly responding tumors are comprised of cells that include 3 lines that are in the S radiosensitivity group: HCT-1116 (wtTP53) and two sublines abrogated in TP53 (379.2) and 14-3-3σ (14-3-3σ -/-). It also includes one cell line from the R radiosensitivity group DLD-1 (mutTP53). Even though S cells in general are more sensitive than R cells in vitro, representatives of both groups fall into the same, relatively linearly responding tumor radiosensitivity group. We will examine this relationship in more detail below.

The patterns of tumor radiosensitivity in the right hand panel of figure 4 are significantly different, showing a more sensitive response, especially at higher doses and larger fraction sizes. While we previously documented this increased response in 80S4 cells (wtTP53 p21-/-), increased sensitivity of other three cell lines; 19S186 cells (mutTP53 p21-/-); SW1222 cells (mutATM) and U251 cells (glio) was unexpected, especially U251 which is a very resistant glioblastoma cell line based on in vitro radiosensitivity. We interpret the data in the right hand panel of figure 4 to demonstrate a heretofore undocumented mechanism that renders some tumors significantly more sensitive to radiotherapy. For the purpose of discussion we will designate these as the p21- tumor radiosensitivity group since all cell show diminished expression of p21 (table 1). In the p21- tumor radiosensitivity group there is a strong effect observed at higher dose-fractions, particularly 15 Gy. We emphasize that this designation does not imply necessarily that p21 is directly involved in tumor radiosensitivity although this relationship needs further investigation.

### A quantitative model for the relationship between tumor response and total dose

The data in figure 4 can be expressed as a relationship between observed tumor response and logs of total-cells- killed, but this relationship is clearly different between tumor cells in the left hand panel and right hand panel. Therefore the overall relationship between tumor responses described in Modal Specific Growth Delay to total dose is not a simple linear relationship but must be expressed in terms of at least two factors that influence quantitative variation across genotype, fraction size and total dose.

After considerable preliminary calculations we propose to define a direct relationship between tumor response and total dose related by two coefficients that represent separately the effects of in vitro radiosensitivity and in vivo radiosensitivity. In general terms this would state that tumor response (TR) would be equal to total dose modified by two coefficients, **τ **that is an estimate of relative sensitivity in vitro and **ρ **that is an estimate of additional radiosensitivity observed in vivo. This equation is shown below:

(1) TR(G,d,nd)=ρ(G,d)×τ(G,d)×D(nd)

In equation 1, TR (tumor response) is expressed in days of modal specific growth delay (mSGD) and is a function of genotype **G**, total dose **nd **delivered in fractions size **d**. The two modifying coefficients **τ **and **ρ **vary with genotype and fraction-size. The factor **τ **represents in vitro radiosensitivity expressed as the ratio of total-cells-killed in vitro per unit dose. The factor **ρ **represents a coefficient that expresses additional in vivo radiosensitivity that cannot be accounted for by cell killing. We emphasize that the relationship in equation 1, is specific to genotype, fraction size and total dose as indicated by subscripts.

### Calculating coefficients that relate tumor response and total dose on the basis of phenotype

**Figure 5:**
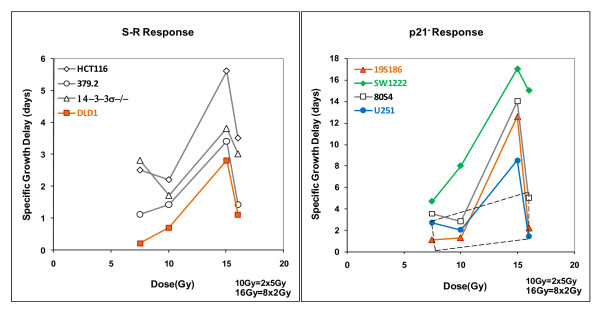
**Total-cells killed expressed as logs_10 _of surviving fractions for eight cell lines treated with protocols of 1 × 7.5 Gy, 1 × 15 Gy, 2 × 5 Gy and 8 × 2 Gy and plotted as total dose for each protocol**. Data in the left panel shows four cell lines hypothesized to express a common "S-R tumor response phenotype". Data in the right panel shows the other four cell lines 19S186, SW1222, 80S4 and U251 that are cell lines that have diminished expression of p21. Error bars are derived directly from survival patterns in figure 1.

We calculated the coefficient **τ **in equation 1 as total-cells-killed per Gy in vitro from the survival data in figure 1 in two steps. In figure 5 we show the relationship between total-cells-killed as a function of total dose for the eight genotypes.

These patterns are a direct portrayal of the changes in cell killing for the four protocols derived from the survival curves in figure 1.

**Figure 6:**
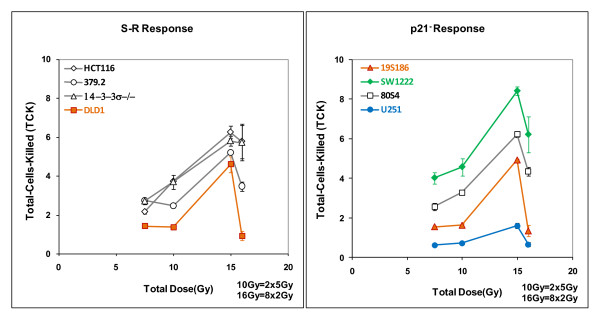
**Tumor response compared to total dose for eight cell lines and four radiotherapy protocols**. Specific Growth Delay in days is compared to total dose delivered for the entire protocols. Tumors were irradiated either with two doses delivered as a single fraction (7.5 or 15.0 Gy) or with two fractionated regimens (2 fractions of 5 Gy each or 8 fractions of 2 Gy each). Responses to acutely delivered single fractions are connected by solid lines; responses to fractionated protocols are connected by dotted lines for each tumor type. The scales are different in the two panels and all data in the left hand panel falls within the dashed trapezoid shown in the right hand panel.

These data show a general overlapping for the two groups of genotypes. In a similar manner, tumor growth delay can be shown as a function of total dose and we show this in figure 6, the data derived from figure 4.

These data show significant differences in vivo radiosensitivity between the two groups of tumor genotypes. The major differences are at higher doses and for single fractions.

**Figure 7:**
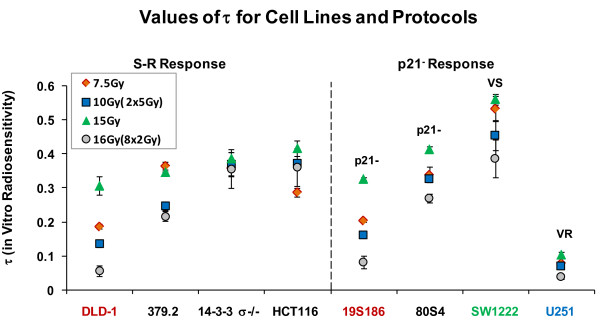
**Values for the parameter τ (logs of total-cells-killed per Gy) for each radiation protocol for each of the eight cell genotypes**. The left panel shows the four cell lines we hypothesize to be the S -R tumor response group and the right panel shows the other four lines.

From figure 5 we calculated the coefficient **τ **as the ratio of modal specific growth delay and total dose. These data are shown in figure 7.

In this set of cells and protocols, **τ **varies between cell lines up to a factor of ~12 (U-251 versus SW1222) and between different protocols in a single cell line up to a difference of up to a factor ~6 (DLD-1 cells, 15 Gy acute versus 8 × 2 fractionated).

**Figure 8:**
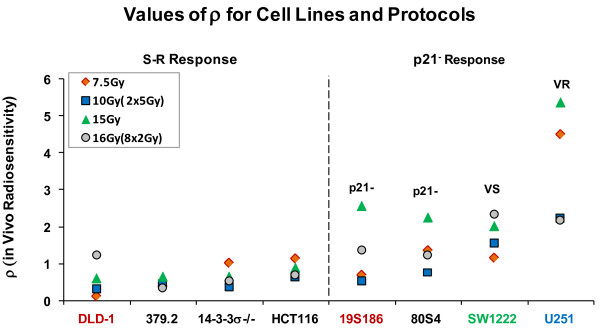
**Values for the parameter ρ (mSGD/logs of cells killed) for each of the eight cell lines for each of the five protocols**. Left panel shows the four cell lines we hypothesize to be the S-R tumor response group and the right panel shows the other four cell lines that have significantly elevated response to 15 Gy. Data represent individual estimates.

In a similar manner we calculated the parameter **ρ **from the data in figure 6 and these data are shown in figure 8.

The data in figure 8 represent additional tumor response per Gy for observed tumor response for the eight genotypes and four radiation protocols. The data in figure 8 show remarkably similar values of **ρ **for the S-R response group over all doses but elevated levels for the p21- response (80S4 and 19S186) only for single doses of 15 Gy. Elevated levels for all responses for the VS response (SW1222 cells); and surprisingly, much elevated values for the VR response (U251 cells). When the 16 values of ρ for the S-R responses are compared to the 16 values of cells from the other response groups there is a highly significant difference (p < 0.005).

### Tumor responses in vivo analyzed as combined effects of two genotype-dependent coefficients that determine tumor response

**Figure 9:**
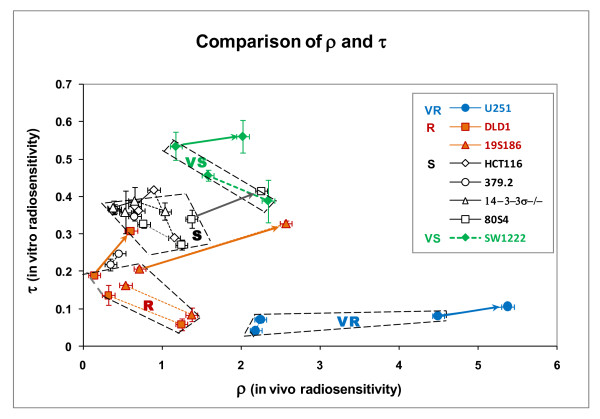
**Comparison of parameters that describe in vitro radiosensitivity (τ) and in vivo radiosensitivity (ρ) for each cell line and irradiation protocol**. Lines connect the same pairs of response points for each cell line as shown in figure 3, where for each cell type solid lines connect the two acute protocols and dashed lines connect the two fractionated protocols. The heavy arrows indicate the increase in response for 15 Gy compared to response to 7.5 Gy. Error bars represent standard error of the mean for values of τ and ρ derived from figures 6 and 7.

The patterns for variation in **τ **and **ρ **(figure 7 and figure 8) define clustering of tumor response on the basis of genotype. These variations are more clearly seen when values of **τ **and **ρ **are plotted against each other for each genotype and for each protocol. This comparison is shown in figure 9.

Data in this figure resolve tumor response into multiple, distinct clusters of data based on the parameters **τ **and **ρ**. Heavy arrows identify the pronounced increase response to 15 Gy for five cell lines. Four tumor response groups are identified and these correspond to the four in vitro radiosensitivity groups identified in figure 1. In figure 9 these groups are further defined on the values of the parameters τ and ρ. The S and R response groups share similar values of ρ but are statistically different based on τ. A VS response group is defined by significantly increased values for both τ and ρ. The VR group is defined by significantly lower values of τ than all other cell lines but highest values of ρ. Two data points for 379.2 cells (abrogated TP53) fall between the S and R groups. The four groups are statistically different based on t-test analyses with p < 0.05 as the criterion for significance.

### Predicting tumor response on the basis of genotype and fraction size

**Figure 10:**
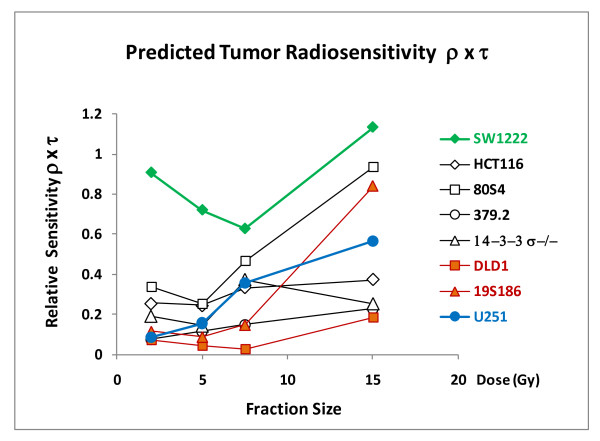
**The relative effect of fraction size on relative sensitivity to tumor response for the eight genotypes**. The ordinate is the product of ρ × τ and represents the overall relative tumor radiosensitivity.

To illustrate how these parameters can be used to predict tumor response for specific genotypes and specific protocols we plot relative sensitivity to radiotherapy as the product of the two coefficients and this is shown in figure 10 as a function of fraction size.

These data demonstrate that tumor response varies strongly based on fraction size and tumor cell genotype.

### Predicting genotype-dependent variation in response to tumor radiotherapy to different protocols

**Figure 11:**
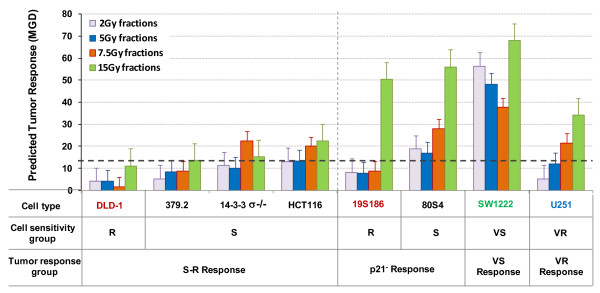
**Predicted response of tumors comprised of the eight tumor cell lines to four different radiotherapy protocols that would deliver 60 Gy: 30 fractions of 2 Gy; 12 fractions of 5 Gy; 8 fractions of 7.5 Gy; or 4 fractions of 15 Gy**. Tumor response was calculated by multiplying ρ and τ for each cell line for each protocol by 60 Gy. The legend at the bottom of this figure represents our hypothesis for the relationships between cell type, cellular radiosensitivity groups and tumor response groups.

Equation 1, once the coefficients **ρ **and **τ **have been defined, can also be used to predict the response of tumor cells to any protocol. In figure 11 we show such predictions for the eight genotypes studied to four hypothetical protocols that all deliver 60 Gy: 30 × 2Gy; 12 × 5 Gy; 8 × 7.5 Gy and 4 × 15 Gy. These predicted responses are shown in figure 11.

These data demonstrate genotype-dependent variation in predicted tumor response. The extent of this variation based on genotype and fraction-size has a range of approximately 14, a remarkable difference for protocols that deliver the same total dose. The schema at the bottom of this figure identifies the relationship between in vitro cellular radiosensitivity groups and in vivo tumor radiosensitivity groups.

## Discussion

Our studies demonstrate three major observations.

1) We have established a major data base comparing radiosensitivity in vitro and xenograft tumor response in vivo for human tumor cells that represent the range of human tumor cell radiosensitivity. These data segregate with tumor cell genotypes that become markers for in vivo radiosensitivity response groups.

2) We have developed an analytical structure that predicts response of tumors to different protocols based on tumor cell genotype and fraction size. This structure separates the effects of genotype, fraction size and total dose on tumor radiosensitivity. This structure is based on defining two coefficients, each representing different independent mechanisms. One coefficient defines TCK (total cell killing (TCK) as measured in vitro. The second coefficient defines additional effects in vivo that are not predicted by TCK. Both coefficients vary with genotype and fraction-size.

3) We have defined a heretofore unreported mechanism of in vivo radiosensitivity that is dependent on tumor cell genotype and fraction size. This mechanism can dominate tumor response but is only observed in some genotypes and for larger fractions.

The data in figures 1, 2 and 3 define groups of tumor cells that share in vitro radiosensitivity and share expression of specific genotypes. We have used this variation in tumor cell radiosensitivity to define four radiosensitivity groups. Our analysis of tumor response as it relates to genotype now allows us to define "tumor response groups". These groups are represented by their values of **τ **and **ρ **and are listed below.

1) S (wtTP53) tumor response: This tumor response is represented in this paper by cell lines from the S radiosensitivity groups that express wtTP53. We have identified 17 cell lines that share this radiosensitivity group and each expresses wtTP53 [3]. Cells abrogated in TP53 [2,3] also fall into this radiosensitivity group and hence the radiosensitivity of this group does not reflect the direct contribution of the wtp53 protein. In figure 11 we use the response of one of these cell lines, HCT116 to 60 Gy delivered as 2 Gy fractions as a basis for comparison for other genotypes and other fraction sizes.

2) R (mutTP53) response. This response is represented by cell lines from the R radiosensitivity group that express predominantly but not exclusively, mutTP53. We have identified 14 cell lines that share this cellular radiosensitivity [3] and together with the S radiosensitivity group these two radiosensitivity groups represent over 90% of human tumor cell lines that we have examined. These cell lines share a common value of **ρ **with the S tumor response group indicating similar in vivo mechanisms of response but differ in values of **τ **directly related to their decreased radiosensitivity in vitro.

2) VS (mutATM) tumor response: This response is observed in xenograft tumors comprised of a single cell line (SW1222) which is mutated in the ATM gene. We have shown SW1222 cells to be extremely radiosensitive and show dramatically increased expression of apoptosis and dysfunctional progression in the cell cycle [21]. The VS response is characterized by the highest values of **τ **(in vitro radiosensitivity) of cells but also shows an increase in response in **ρ **compared to S and R responses. Additionally, this response includes enhanced sensitivity to a single large fractions (15 Gy), a property shared with other cell lines that express diminished p21.

3) VR ("glio") tumor response. The VR group is represented by a single cell line in these studies, the radioresistant glioblastoma cell line U251. We predict, however, that other radioresistant cell lines that share extreme in vitro radioresistance may share this tumor response [2,3]. Tumors comprised of U251 cells are characterized by the most resistant intrinsic cellular sensitivity in vitro (smallest **τ**) we have observed but are characterized, surprising to us, the highest values of **ρ **for any cell line, particularly for larger single fractions. If this is a characteristic of all radioresistant glioblastoma lines, it not only suggests a rationale for large-fraction therapy of brain tumors but also offers a more precise clinical strategy for attacking this class of tumors. We caution, however, that our studies were performed with tumors implanted in the flanks of nude mice. We do not know whether glioblastomas within the cranium would respond in a similar way.

4) p21^- ^tumor response to larger fraction-size: This response is observed in five cell lines and is characterized at the cellular level by diminished expression of p21 (DLD-1, 19S186, 80S4, SW1222 and U251) and at the tumor level by common enhanced sensitivity only to large fractions (15 Gy). Note that in our overall classification, we place both DLD-1 and its subline 80S4 that is abrogated into R cellular radiosensitivity group and both exhibit increased response to 15 Gy, but the relative response of the line abrogated in p21 results into increased sensitivity of this subline similar to the exaggerated response to 15 Gy by the S cell line abrogated in p21, or the other three cell lines in the p21- response group. While only two of these lines are abrogated in the CDKN1A (p21) gene, the other three cell lines show diminished p21 induction as measured by Western blot analysis. Others have reported cells with deficient ATM [22] and radioresistant glioblastoma cells [23,24] express diminished p21 in vivo. We caution that we have certainly not proved that diminished p21 expression in vivo is the mechanism that underlies increased radiosensitivity at higher doses; however it is clearly associated statistically. To us it is an attractive hypothesis but proof will require extensive, detailed assay in vivo in tumors irradiated with smaller and larger doses in which p21 expression, VEGF levels, microvessel density, apoptosis and cell necrosis are measured in p21- tumors compared to genotypes that are competent in the expression of this gene expression.

Our studies define an experimental system that can identify genes that do or do not influence tumor response. Genes that influence tumor response can be described as modifying **ρ **or **τ **or both. The ratio **τ **is influenced by wtTP53, mutTP53, mutATM and glio but not by abrogated p21. The ratio **ρ **is influenced by mutATM, abrogated p21, and glio. Abrogated TP53, abrogated CDKN1A, 14-3-3σ, hMLH2 and hMSH6 do not influence in vitro radiosensitivity as measured in our studies. 14-3-3σ, hMLH2 and hMSH6 do not influence tumor radiosensitivity in vivo.

We have previously hypothesized that differences in **τ **may reflect an influence of chromatin structure on radiosensitivity including access to repair and modulation of apoptosis [1,2] This is supported by several reports: the protective influence of p53 on chromatin structure [25] and chromatin structure as a target for radiation-killing [26]; influence of chromatin structure on repair [27]. We have also previously shown that depletion of polyamines in chromatin sensitizes cells to ionizing radiation delivered at higher doses, Williams et al [28]. Importantly, Hittelman and Pandita [29] have shown radiosensitivity associated with ATM results from an essential difference in chromatin structure that modulates processing of radiation damage. These several reports support the hypothesis that variation in radiosensitivity between the cellular radiosensitivity groups is attributable to changes in chromatin structure that develop during oncogenesis.

In vivo radiosensitivity, we hypothesize, reflects changes in **ρ **observed in the several tumor response groups and may reflect interactions between tumor cell genotype and the tumor microenvironment. We do not know what mechanism underlies the relationship between diminished p21 (p21- response); enhanced cellular apoptosis (VS response); and an enhanced, but unidentified, effect observed in radioresistant glioblastoma cells (VR response). However the differences between in vivo radiosensitivity and p21 expression can be studied using the model systems we have developed. Interestingly, Kuljaca et al [30] have shown that p21 promotes angiogenesis so the interaction of p21 with angiogenesis at higher doses needs further study. Importantly, the creation of an appropriate tumor microenvironment that would enhance tumor response may be achievable using chemical or biological agents.

We make the following overall hypothesis for the relationships between tumor cell genotype, intrinsic cellular radiosensitivity and tumor radiosensitivity:

Tumor cell genotype, used in the broadest sense to include chromatin conformation, correlates with: 1) intrinsic cellular radiosensitivity as defined by clonogenic survival in vitro; and 2) enhanced tumor response in vivo for some genotypes at higher doses reflecting an interaction between tumor cell genotype and the tumor microenvironment. These two mechanisms act independently but together can predict tumor response to different radiotherapy protocols based on tumor cell genotype, fraction size and total dose.

## Abbreviations

Radiosensitivity groups: VS: very sensitive; S: sensitive; R: resistant and VR: very resistant. Tumor response groups: S +R: cells that express most common form of in vivo radiosensitivity. P21- cells exhibit increased in vivo radiosensitivity and all are deficient or reduced in expression of p21. TCK: total-cells-killed; mSGD: modal specific growth delay.

## Competing interests

The authors declare that they have no competing interests.

## Authors' contributions

JW was responsible for overall planning, execution and interpretation of the studies. HZ and YZ performed all studies, recorded and maintained data records. CK and JBL were members of the external advisory committed and worked with JW in planning and interpreting the studies. JFD confirmed dosimetry and treatment planning and contributed to data analysis. DG and JS contributed to interpreting the studies with larger fractions that are achievable with proton therapy. All authors read and approved the manuscript.
